# Trichomonads in the oral cavity of dogs and cats: species composition and relationship with animal age and oral hygiene status

**DOI:** 10.1007/s11259-026-11565-x

**Published:** 2026-10-07

**Authors:** Agnieszka Piekara-Stępińska, Albert Czerski, Barbara Szczepankiewicz, Agnieszka Lachowicz-Wolak, Michał Gorczykowski, Jolanta Piekarska

**Affiliations:** 1 Department of Internal Medicine and Clinic of Diseases of Horses, Dogs and Cats, Division of Parasitology and Invasive Diseases, Faculty of Veterinary Medicine, Wrocław University of Environmental and Life Sciences, 31 Norwida St, Wrocław, 50-375 Poland; 2 Department of Biostructure and Animal Physiology, Division of Animal Physiology, Faculty of Veterinary Medicine, Wroclaw University of Environmental and Life Sciences, Grunwaldzki Sq. 45, Wrocław, 50-366 Poland; 3 Department of Epizootiology and Clinic of Birds and Exotic Animals, Division of Avian, Exotic, Fur and Laboratory Animal Diseases, Faculty of Veterinary Medicine, Wroclaw University of Environmental and Life Sciences, Grunwaldzki Sq. 45, Wrocław, 50-366 Poland; 4 Department of Epizootiology and Clinic of Birds and Exotic Animals, Division of Infectious Diseases of Animals and Veterinary Administration, Faculty of Veterinary Medicine, Wroclaw University of Environmental and Life Sciences, Grunwaldzki Sq. 45, Wrocław, 50-366 Poland

**Keywords:** Dental calculus, Periodontitis, Protozoa, *Trichomonas tenax*, Zoonosis

## Abstract

The oral cavity provides a suitable environment for various microorganisms, including protozoa such as trichomonads, which may be associated with periodontal disease development. Humans are considered the typical hosts of *Trichomonas tenax* but it has also been found in dogs, cats, and other animals, which makes it a factor with a significant zoonotic potential. Apart from *T. tenax*, domestic carnivores may be infected with such species as *T. canistomae*,* T. felistomae* and *T. brixi.* The aim of this study was to determine the prevalence, species composition, and risk factors of trichomonad colonization of the oral cavity in dogs and cats from Poland. Oral swabs from 238 household animals (151 dogs and 87 cats) were examined using cultivation methods. Amplification of ITS1– 5.8 S rRNA-ITS2 fragment was performed for microscopic-positive samples and PCR products were further sequenced. Trichomonads were identified in 26 out of 151 (17.22%) canine and 5 out of 87 (5.75%) feline samples. Higher frequency was observed in older dogs and dogs with intense dental calculus. *T. brixi* and *T. tenax* were the only species detected in both dogs and cats. The detection of zoonotic *T. tenax* in a substantial number of examined animals highlights the relevance of this finding also in the context of human health.

## Introduction

Trichomonads are widespread protozoa found in different locations in a broad spectrum of hosts. They have been detected, among others, in the oral cavity of humans and many animal species, including dogs and cats (Dybicz et al. 2018; Nor Azmi et al. 2024; Matthew et al. 2025). The species typical for dogs and cats are *Trichomonas canistomae/felistomae* or recently described *T. brixi*, but the zoonotic species *T. tenax* has also been found (Kellerová and Tachezy 2017). Pathogenicity of oral trichomonads is widely discussed – they are often considered to be non-pathogenic but numerous studies confirm their association with periodontal diseases (Patel et al. 2017; Kadhim et al. 2023; Matthew et al. 2023; Stoyanov et al. 2024). It has been proven that they are capable of producing hemolysins and proteases that damage collagen fibers and promote soft tissue degradation (Nagao et al. 2000; Yamamoto et al. 2000). They have also been detected outside the oral cavity, in the submandibular salivary glands, tonsils, and lymph nodes. Moreover, they have been associated with pulmonary diseases and the formation of abscesses in the lower respiratory tract, particularly in immunocompromised individuals or those with coexisting diseases (Szczepaniak et al. 2016; Kellerová and Tachezy 2017; Dybicz et al. 2018; Duboucher et al. 2000; Hong et al. 2024).

Despite clear evidence of their role as pathogens, trichomonads remain poorly understood in dogs and cats. These domestic animals are particularly important due to their close contact with humans, which increases the risk of transmission of pathogens with a zoonotic potential. The aim of this study was to determine the prevalence and species composition of trichomonads in the oral cavity of dogs and cats from Poland, as well as the influence of animal age, oral hygiene status, and living environment on the pathogen prevalence.

## Materials and methods

### Materials

The oral swabs were collected between June 2024 and December 2025 from 151 domestic dogs and 87 cats from different regions of Poland (Lower Silesia, Greater Poland, Lodzkie Voivodship, Podlaskie Voivodeship, Pomerania, West Pomeranian Voivodeship, Lubusz Voivodeship, Masovian, Silesia, Subcarpathian) during routine diagnostic and preventive procedures. The study included animals belonging to various breeds. The samples were taken individually from the surface of gingiva and tooth using sterile swabs, placed in the AMIES transport medium (Deltalab, Spain), and delivered to the laboratory at the Division of Parasitology and Invasive Diseases, Wroclaw University of Environmental and Life Sciences within a maximum of 24 h after collection. During transport, the samples were protected from temperature fluctuations by using insulated packaging. The animals were divided into groups according to age (0–4 years old, 5–8 years old, and ≥ 9 years old) and severity of dental calculus (Grade I – lack of dental calculus or up to 25% of the dental surface covered by tartar, Grade II – 25 to 50% of the dental surface covered by tartar, Grade III – over 50% of the dental surface covered by tartar). With regard to age, data were obtained for 134 dogs and 81 cats. The severity of dental calculus was recorded in 141 dogs and 78 cats.

### In vitro culture

The oral swabs were immediately transferred after delivery from the transport medium to sterile falcon tubes containing a modified Pavlova medium (Pavlova 1938) (PBS, 10% of horse serum, rice starch, and penicillin – streptomycin; pH 6.8), cultivated for 48 h at 37º C and then passaged to a fresh medium. A drop of each culture was examined after each passage using a light microscope (ZEISS Axioscope) to detect the presence of trichomonads (based on their characteristic shape, flagella, and motility). This assessment was performed only to confirm the presence of trichomonads and was not used for species identification. Passaging was performed only when no trichomonad growth was observed. When trichomonads were detected by microscopic examination, the sample was considered positive and further passages were not required. The samples, in which trichomonad growth was observed were stored at − 20 °C for further analysis.

### DNA extraction and amplification

DNA extraction was conducted from trichomonads multiplied in the culture (only from the samples showing trichomonad growth) using the Extractme Genomic DNA KIT (BLIRT S.A, Poland) according to the manufacturer’s instruction. The isolates were stored at 4°C and examined within 48 hours. To confirm the presence of trichomonad genetic material and to identify the species, the ITS1– 5.8S rRNA-ITS2 fragment was amplified using T1 (5’-GAGAAGTCGTAACAAGGTAACG-3’) and T2 (5’-ATGCTTCAGTTCAGCGGGTCT-3’) primers (Genomed, Poland) (Dybicz et al. 2018). PCR amplification was performed using a Bio-Rad T100™ Thermal Cycler, and the PCR conditions were as follows: initial denaturation at 94 °C for 3 min, followed by 30 cycles of denaturation at 94 °C for 30 s, annealing at 50 °C for 1 min, and extension at 72 °C for 1 min, with a final extension at 72 °C for 7 min. The PCR products were examined electrophoretically in 2% agarose gels and visualized after staining with Midori Green Advance DNA Stain (Nippon Genetics Europe GmbH, Düren, Germany). The expected product size was approximately 370 bp.

### DNA sequencing and data analysis

The PCR products were purified and sequenced in both directions by Genomed (Poland). The chromatograms were visually assessed to exclude the presence of double peaks. Sequence analyzing was performed using BioEdit software (version 7.2.5.0). The obtained sequences were compared by a blast search (https://blast.ncbi.nlm.nih.gov/Blast.cgi) with the sequences deposited in the GenBank.

### Statistical analysis

Statistical analyses were performed using Microsoft Excel and the online statistical calculator VassarStats (http://vassarstats.net/). For each group, the proportion of positive results was calculated, and 95% confidence intervals for proportions were estimated using the Wilson method. The strength of the association between variables was assessed using odds ratios (OR), with the first group treated as the reference category. In cases of zero cell counts in contingency tables, the Haldane–Anscombe correction (+ 0.5) was applied. Differences in the prevalence of trichomonads infections between the study groups were evaluated using the chi-square (χ²) test, with a significance level set at *p* ≤ 0.05. In the case of zero cell counts, Fisher’s exact test was used (reporting a two-tailed p-value), or, for 2 × 3 tables, its Freeman–Halton extension.

## Results

Oral trichomonads were detected significantly more frequently in dogs than in cats (*p* < 0.05). In vitro cultivation showed the presence of trichomonads in 26 out of 151 (17.22%; 95% CI, 11.75%–24.4%) canine and 5 out of 87 (5.75%; 95% CI: 2.13%–13.5%) feline samples. The PCR analysis confirmed the presence of trichomonad genetic material in all microscopically positive samples. In dogs, the highest prevalence was observed in older animals (over 9 years old) and individuals with severe dental tartar deposition (with over 50% of the dental surface covered with calculus), the differences were statistically significant. There were no significant differences associated with age, living environment, and oral hygiene status in cats. Details are presented in Table 1.

**Table 1:** Detailed data on the occurrence of oral trichomonads in the individual groups

Species	Group size	Groups	total	No of positive samples	Percent of positive samples (%)	95% (CI)	OR	*p*
Dogs	134	Age						
		0–4	46	4	8.69	2.82–21.69		*p* = 0.049*
		5–8	39	8	20.51	9.87–36.94	2.71	
		> 9	49	14	28.57	17.03–43.47	4.20	
	141	Severity of the tartar						
		Grade I	66	5	7.58	2.82–17.50		*p* = 0.0004*
		Grade II	28	4	14.29	4.68–33.56	2.03	
		Grade III	47	17	36.17	23.06–51.52	6.91	
Cats	81	Age						
		0–4	36	3	8.33	2.18–23.59		*p* = 0.5963
		5–8	25	2	8.0	1.40–27.5	1.02	
		> 9	20	0	0	0.00-20.04	0.23	
	78	Severity of the tartar						
		Grade I	50	3	6.0	1.56–17.54		*p* = 0.4131
		Grade II	13	0	0	0.00-28.34	0.50	
		Grade III	15	2	13.33	2.34–41.61	2.51	
	80	Living environment						
		outdoor	12	0	0	0.00-30.12		*p* = 0.5935
		indoor	68	5	7.35	2.74–17.02	2.17	

^*^ statistically significant value

In dogs, 23 out of 26 positive samples were successfully sequenced, and the most frequently identified species was *Trichomonas brixi* (65.2%, 15 out of 23 sequenced samples), followed by *Trichomonas tenax* (34.8%, 8 out of 23 sequenced samples). In cats, the sequencing identified *T. tenax* (80%, 4 out of 5 samples) and *T. brixi* (20%, 1 out of 5 samples). Nucleotide sequence data reported in this study have been deposited in the GenBank database with accession numbers PZ658394-PZ658421. Detailed data are provided in Table 2.

**Table 2:** Detailed data on the species composition of oral trichomonads in positive samples

Sample ID and GenBank accession number	Closest matching sequences from GenBank	Identity (%)
Dog18 PZ658394	*Trichomonas brixi* KX977526.1	100
Dog39 PZ658395	*Trichomonas brixi* KX977521.1	100
Dog43 PZ658396	*Trichomonas brixi* KX977521.1	100
Dog52 PZ658397	*Trichomonas brixi* KX977521.1	100
Dog53 PZ658398	*Trichomonas tenax* PX602343.1	100
Dog68 PZ658399	*Trichomonas brixi* KX977521.1	100
Dog75 PZ658400	*Trichomonas brixi* KX977521.1	100
Dog76 PZ658401	*Trichomonas brixi* KX977521.1	100
Dog77 PZ658402	*Trichomonas tenax* KX977536.1	100
Dog80 PZ658403	*Trichomonas brixi* LC710808.1	99.50
Dog85 PZ658404	*Trichomonas brixi* PX602352.1	99.69
Dog86 PZ658405	*Trichomonas brixi* PX602352.1	100
Dog91 PZ658406	*Trichomonas tenax* KX186997.1	100
Dog93 PZ658407	*Trichomonas brixi* KX977526.1	100
Dog103 PZ658408	*Trichomonas tenax* PX602343.1	100
Dog106 PZ658409	*Trichomonas tenax* PX602343.1	100
Dog110 PZ658410	*Trichomonas tenax* KX186997.1	100
Dog112 PZ658411	*Trichomonas tenax* U86615.1	100
Dog1Lodz PZ658412	*Trichomonas tenax* KX186997.1	100
Dog1M PZ658413	*Trichomonas brixi* KX977526.1	100
Dog4S PZ658414	Trichomonas brixi KX977521.1	100
Dog17W PZ658415	*Trichomonas brixi* KX977521.1	100
Dog18W PZ658416	*Trichomonas brixi* KX977521.1	100
CatK3 PZ658417	*Trichomonas tenax* KX186997.1	100
CatK8 PZ658418	*Trichomonas tenax* U86615.1	100
CatK30 PZ658419	*Trichomonas tenax* PX602343.1	100
CatK31 PZ658420	*Trichomonas tenax* PX602345.1	100
CatK40 PZ658421	*Trichomonas brixi* KX977521.1	100

## Discussion

Available data on the occurrence of oral trichomonads in carnivores are limited. In Poland, the prevalence was determined to be 4.9% in dogs and 5.3% in cats by PCR (Dybicz et al. 2018). In this study, the prevalence was estimated at 17.22% in dogs and 5.75% in cats. Although the result in cats is comparable to that obtained by Dybicz et al., in dogs it is markedly higher. This may be attributed to shifts in the epidemiological landscape over the past several years. However, it should also be taken into account that differences in the obtained results may have been influenced by factors such as the use of different methods for trichomonads detection and the housing conditions of the individuals included in the study group. In the neighboring Czech Republic, the prevalence assessed by the cultivation method was considerably higher than that observed in our study, reaching 37.8% in dogs and 9.8% in cats (Kellerová and Tachezy 2017). That study included also shelter animals, however, the authors suggest that domestic individuals were at a higher risk of infection than the shelter animals. In a PCR-based study conducted in Hungary, no oral trichomonads were detected in any of the examined samples, although the small study population may have affected the results (Tuska-Szalay et al. 2024). Current epidemiological status of dogs was also assessed in Japan and East China, using PCR method. In Japan, the result was comparable to that of our study, amounting to 14.6%, whereas in the Chinese population a markedly lower proportion of positive individuals (2.8%) was found (Ito et al. 2023; Gui et al. 2025). 

In our study, oral trichomonads were detected significantly more frequently in dogs than in cats (p<0.05). The previous studies also reported similar results (Kellerová and Tachezy 2017). The difference is likely not due to lower susceptibility in cats, but rather to distinct behaviors and different housing conditions. Dogs generally have greater opportunities for contact with other individuals and the external environment, which is associated with a higher risk of infection. Additionally, cats are typically more reserved in interactions with conspecifics and often have limited exposure to the external environment, which decreases the chance of contact with pathogens (Menchetti et al. 2020). 

In the present study, we observed that prevalence of oral trichomonads in dogs increased with age, reaching its highest level in the group of animals older than 9 years. Higher frequency was also associated with poor oral hygiene status and severity of the dental calculus. These factors may be associated with changes in the oral microenvironment, including pH alterations and the development of sites with limited oxygen availability, which could promote colonization by trichomonads (Matthew et al. 2025). Regarding age, similar observations have been reported in previous studies (Kellerová and Tachezy 2017). It is worth noting that these two factors may be associated, since the level of oral hygiene in dogs decreases with age (Kortegaard et al. 2008; Hemida et al. 2025). Interestingly, trichomonads were detected only in indoor cats in this study, although the difference between indoor and outdoor animals was not statistically significant.

In our study, dogs and cats were infected by two species: *T. tenax* and *T. brixi*, but at different rate. In dogs, *T. brixi *was found to be more common than *T. tenax* (65.2% vs 34.8% of the sequenced samples), whereas an opposite pattern was observed in cats (80% *T. tenax* vs 20% *T. brixi*). The housing conditions seem to play a significant role again, as all cats in which oral trichomonads were detected were indoor animals, with limited contact with the external environment but closer contact with humans. Therefore, the primary source of infection seem to be humans, for whom *T. tenax* is considered a typical species. However, this is only a hypothesis, since samples were not collected from the owners, and therefore this assumption cannot be unequivocally confirmed. *T. brixi *is a relatively recently described species, and our study confirmed its presence in Poland for the first time. Interestingly, the studies from the Czech Republic and Japan also showed it as the most frequent trichomonad in dogs (Kellerová and Tachezy 2017; Ito et al. 2023). So far, *T. brixi* has not been found in humans and it seems typical for dogs and cats. Three dog samples could not be successfully sequenced. The obtained sequences were of too low quality (due to the lack of reliable base calls) to enable identification of the trichomonads species. Poor sequence quality may result from insufficient DNA quantity or integrity, as well as from technical limitations during the sequencing process. However, the exact cause of the observed sequencing failure in this study could not be determined.

It is worth noting that, according to the available literature, although Pavlova medium is not currently the most commonly used medium for trichomonad cultivation, it is also applied and supports the growth of these protozoa (Tasca et al. 2001; Al-Dabbagh and Kharofa 2007). Tasca et al. (2001) reported that the medium did not allow for accurate microscopic observation of the trichomonads due to the presence of visible starch granules and bacterial growth. We did not encounter a similar problem in our study, however, the purpose of the microscopic examination was to determine the presence or absence of trichomonads and to facilitate their multiplication for subsequent molecular analyses, rather than to assess their detailed morphological features.

In conclusion, this research showed the current prevalence and species composition of oral trichomonads in dogs and cats from Poland. The study confirmed the presence of the zoonotic *T. tenax* and demonstrated the presence of *T. brixi* for the first time in dogs and cats in Poland. Moreover, the significance of risk factors, such as age or oral hygiene status in dogs was confirmed. Our results update and supplement the research findings in this field and may be important for both animal and human health.

## Data Availability

All essential data are included in the manuscript. The sequences obtained in this study have been deposited in GenBank database. Additional data can be provided upon request by contacting the corresponding author via email.
